# Reinforcement Learning and Decision Making in Anorexia Nervosa

**DOI:** 10.1007/s11920-025-01643-3

**Published:** 2025-10-07

**Authors:** Christina E. Wierenga, Carina S. Brown, Erin E. Reilly

**Affiliations:** 1 University of California San Diego, Department of Psychiatry, San Diego, CA USA; 2 San Diego State University/University of California San Diego Joint Doctoral Program in Clinical Psychology, San Diego, CA USA; 3 University of California San Francisco, Department of Psychiatry and Behavioral Sciences, San Francisco, CA USA

**Keywords:** Anorexia nervosa, Decision-making, Eating disorders, Reinforcement learning, Reward, Punishment

## Abstract

**Purpose of Review:**

We review recent literature on instrumental reinforcement learning involving decision-making in anorexia nervosa (AN) to understand mechanisms underlying symptoms of AN, such as rigid pursuit of weight loss despite negative consequences.

**Recent Findings:**

Relatively consistent findings indicate worse reward- and punishment-based feedback learning in the ill and weight-recovered states that is not observed in remitted samples. Initial studies suggest decreased goal-directed learning in AN, although this needs replication. Similarly, research is needed to clarify mixed findings related to learning under changing rules and the role of fear versus avoidance learning in AN.

**Summary:**

Growing evidence supports altered reinforcement learning in AN. Most studies examined the impact of outcome valence, changing rules, and habitual vs goal-directed control on learning. Computational modeling approaches can provide nuanced characterization of cognitive processes related to reinforcement learning and contribute to precision medicine efforts that may improve outcomes.

## Introduction

Anorexia nervosa (AN) is a serious and medically-dangerous psychiatric disorder characterized by extreme restrictive eating and significant weight loss, an intense fear of weight gain, and disturbed body-related experience [1]. It is associated with increased mortality risk [2], medical complications [3], psychiatric comorbidity [4], functional impairment [5], and poor treatment response, often resulting in protracted illness [6]. Evidence-based psychotherapies for AN are associated with symptom abstinence in 30–60% of treatment-seekers [7], and relapse following treatment completion is common [8]. Suboptimal treatment effects call into question whether key aspects of eating disorder psychopathology are being effectively addressed by current interventional approaches. A better understanding of neurobiological mechanisms driving AN symptoms may improve treatments. Altered reinforcement learning and value-based decision-making, which relate to symptoms of many psychiatric disorders, including anxiety and depression [9, 10], may be implicated in core AN features, such as persistent dietary restriction and compensatory weight loss behaviors (e.g., purging) despite their negative consequences [11–14]. Further, learning alterations are known to impact response to standard psychological therapies in other disorders [15, 16], suggesting that characterization of reinforcement learning in AN may improve outcomes.

The ability to flexibly learn from experience, based on learning to maximize rewarding outcomes and to avoid aversive outcomes, known as reinforcement learning, is required to adapt to changing environments. Reinforcement learning theories focus on the impact of learned associations between stimuli (e.g., cues), behavioral responses (e.g., automatic responses or actions performed), and outcomes (e.g., positive or negative feedback) on behavior and are based on the notion that in general, rewarded behavioral responses will be repeated and punished behavioral responses will be avoided [17]. Core theories of reinforcement learning hypothesize learning rates are modulated by expectancy violations, or prediction errors (PE), which reflect the difference between received and expected outcome and are associated with dopamine activity in the striatum [18, 19]. Within this framework, positive PEs (outcomes better than expected) increase dopamine firing, negative PEs (outcomes worse than expected) decrease dopamine firing, and expected outcomes maintain a tonic level of firing [20, 21]. Dopamine signaling modulates corticostriatal plasticity, and differences in phasic dopaminergic signaling can strengthen action selection pathways driving behavior [20, 22]. Learning from experience occurs through updating expectations about the outcome in proportion to PE, such that, over time, expected outcomes should converge to the actual outcomes.

Pavlovian or classical conditioning involves stimulus-outcome learning related to automatic or conditioned behavioral response (e.g., salivating) to a cue (e.g., bell) as a result of learned expectations about cue-outcome pairings (e.g., food). On the other hand, during instrumental or operant learning, outcomes (e.g., weight loss) are contingent on learned actions (e.g., dietary restriction) in response to a cue (e.g., food), involving stimulus-response (i.e., habit) and response-outcome (i.e., associative) learning. Existing frameworks for defining goal-directed and habitual behaviors outline that while goal-directed action includes decisions that are flexibly implemented based on anticipated consequences (i.e., outcomes) and able to shift rapidly in response to environmental changes, habitual actions are formed through repetition, are automatically engaged in response to a stimulus, and are less sensitive to outcome and slower to update when the environment changes [23, 24]. Research has suggested that these different aspects of reinforcement learning may contribute to repeated engagement in maladaptive behavior, such as rigid pursuit of thinness in AN [25–28].

Increasing empirical evidence supports the importance of choice behavior and decision-making in the emergence and maintenance of psychiatric disorders marked by inflexible or excessive goal pursuit, including AN; see [12, 26, 29–31] for conceptual and systematic reviews. In the context of reinforcement learning, decision-making is influenced by stimulus valuation (e.g., appraisal of reward/threat) and requires cognitive flexibility to update information, modify behavior according to potentially changing states, and arbitrate between options to guide choices in pursuit of short- or long-term goals [32, 33]. This process also involves exploration of new options versus exploitation of gained knowledge. Individuals learn whether a decision was consistent with their goal based on the outcome.

Reinforcement learning and decision-making frameworks have informed conceptual models for understanding the onset and maintenance of eating pathology in AN. AN symptoms such as choices about food, caloric intake, or exercise are viewed as value-driven decisions that are learned through a series of rewarding and punishing outcomes [34]. For example, food choices or avoidance may initially develop through receipt of positive outcomes (e.g., weight loss, social praise) or avoidance of negative outcomes (e.g., lack of weight gain) [35]. As an individual learns, they build expectancies based on reinforcement and punishment histories that influence their beliefs about the actions they can take and stimuli that predict different outcomes, shaping and motivating future behavior [36]. Given that most people are exposed to information that could result in maladaptive food- and body-related learning, yet few individuals develop AN, theoretical models have posited that individuals with AN may have unique risk factors (e.g., genetic, neurobiological) that result in this learning happening more readily, becoming overgeneralized, or being more resistant to change [11, 14, 35].

## Evidence of Altered Reinforcement Learning and Decision-Making in AN

As there is growing interest in both the role of learning and decision-making in AN, this review will focus primarily on recent behavioral studies examining instrumental reinforcement learning in AN that involve learning based on the outcome of one’s actions or decisions (e.g., choices). This includes both stimulus-response (i.e., habit) and response-outcome (i.e., associative or goal-directed) learning; for review of classical conditioning in AN, see [11]. Because learning and decision-making are closely related and often dually implicated in existing learning tasks, this review includes tasks that involve both processes; however, we constrain our focus on decision-making to only include those tasks that involve analysis of decisions based on what has been learned through experience (i.e., reinforcement learning). For a broader review of decision-making processes in AN, see [12].

**Fig. 1:**
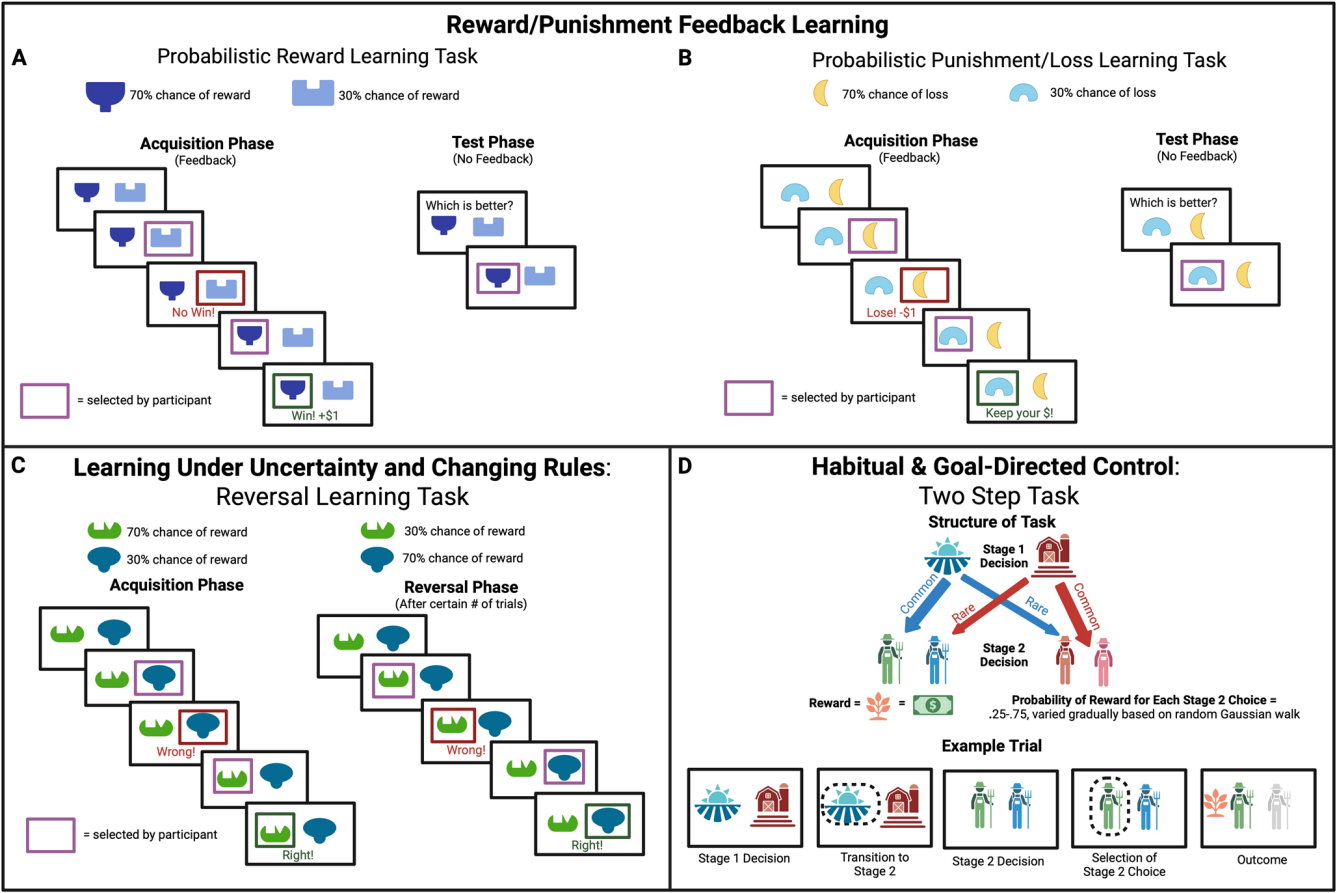
Exemplar paradigms used to measure reinforcement learning. Four task paradigms commonly used to measure reinforcement learning: **(A)** A common task used to gauge learning from rewarding experience. Participants are asked to learn from trial-by-trial probabilistic outcomes regarding which stimulus (or stimuli) is associated with higher probability of reward. Sometimes these tasks may include a test phase, wherein prior learning (and thus, expected values) can be gauged through choices performed in the absence of feedback. **(B)** A probabilistic learning task wherein salient outcomes involve potential loss. Participants are asked to learn which stimulus is associated with greater likelihood of loss through trial-by-trial experience. **(C)** Reversal learning task that prompts participants to learn to select a stimulus that is associated with higher probability of reward; following a change in contingencies (i.e., reversal), participants are required to update their behavior and expected values for stimuli sets. **(D)** A hierarchical reinforcement learning task used to gauge goal-directed and habitual behavior. Participants are asked to select one of two stimuli at stage 1, which are probabilistically associated with being directed to one of two stage 2 decisions. Participants then select a stimulus at stage 2, with each stimulus associated with varying probabilities of reward that vary throughout the task. Participants’ trial-by-trial decisions can be analyzed to determine whether they have learned expected values of decisions based on a higher-order understanding of the task structure (i.e., goal-directed) or based on more rudimentary trial-by-trial outcomes (i.e., habitual)

Studies of reinforcement learning in AN have most frequenly assessed probabilistic associative learning and reversal learning. During probabilistic associative learning, individuals learn relationships between stimuli and actions based on the probability of their co-occurrence (e.g., selecting stimulus A is associated with reward 70% of the time), and these probabilities may differ across stimuli (e.g., selecting stimulus B is associated with reward 80% of the time) or change throughout a task (as in “volatility” or adaptive tasks). Reversal learning, which has been termed “learning to ‘unlearn’”, involves learning to inhibit a previously learned response when outcome contingencies change (i.e., when a previously rewarded action is no longer rewarded or a previously punished action becomes the rewarded action), which requires adapting to new rules or stimulus-outcome associations. Fewer studies have examined learning under conditions of threat, or evaluated the influence of habit vs. goal-directed decision-making on learning, although these represent promising avenues for future work. Thus, we organize our review according to the following themes: (1) studies that assess reward and punishment feedback learning to evaluate the impact of outcome valence on learning, (2) studies that assess learning when there are changing rules to evaluate the impact of uncertainty or change on learning, (3) studies that evaluate fear or avoidance learning to evaluate the influence of threat on learning, and (4) studies that examine the influence of habit (stimulus-response) vs. goal-directed (response-outcome) decisional processes on learning. Paradigms used to measure reinforcement learning are illustrated in Fig. 1.

### Reward and Punishment Feedback Learning in AN

Reinforcement learning by definition is influenced by stimulus valuation. Individuals with AN demonstrate differences in subjective sensitivity to reward and punishment, as well as alterations in reward neural circuitry, raising the possibility that altered stimulus valuation may impact value-based learning [37–39]. To address this question, several studies have examined whether learning from reward and/or punishment is differentially implicated in AN. Studies of feedback-based reward learning in AN tend to show worse performance in acutely ill and weight-restored individuals compared to healthy controls [25, 27, 40–42]. This is consistent with generally lower self-reported and behavioral reward responsiveness and reduced neural response in reward circuitry to food and monetary reward in AN [38, 43–45]. However, such disturbances in reward feedback learning are not consistently observed in individuals remitted from AN [46, 47], suggesting possible illness state effects.

Results from punishment-based feedback learning studies are less consistent in individuals acutely ill, despite the common finding of increased punishment sensitivity in AN [37, 48]. For instance, there is evidence of increased punishment learning during reversal learning in adolescents with AN [49] (see the next section for more detail), but no differences in probabilistic learning from negative feedback in adolescents [41] and slower punishment learning in ill adults [27, 46]. Interestingly, individuals remitted from AN tend to show faster punishment-based feedback learning on tasks that involve changing rules (i.e., reversal learning) and probabilistic associative learning tasks [28, 50]. Notably, while associations between learning from positive or negative feedback and illness-related variables were not detected in adolescent AN [41], we found greater magnitude of negative PE (i.e., worse punishment learning) was associated with less weight gain during treatment in adults. In combination with findings of faster punishment learning in remitted AN, this raises the hypothesis that poorer loss-related learning may contribute to AN persistence.

### Learning Under Changing Rules and Uncertainty in AN

Given known alterations in cognitive flexibility and set-shifting in AN [51, 52], several studies have evaluated the ability to update previously-learned associations following changes in the environment (e.g., changing rules or stimulus-response-outcome contingencies) using reversal learning or volatility learning tasks. These tasks ask the participant to learn initial behaviors or responses, and then, once the environment and contingencies have changed, shift their responses, update previously-learned information, and learn something new. Successful completion of the task is also facilitated from the participant engaging in meta-learning about the larger structure of the task and the volatility of the environment (i.e., accurate prediction of how frequently and in what manner contingencies may change). Performance is typically evaluated with learning rates that are mathematically calculated based on a participant’s observed behaviors over time following receiving negative or positive outcomes, or according to lose-shift behavior (i.e., tendency to change one’s response after receiving a negative outcome) and/or win-stay behavior (i.e., tendency to perform the same response after receiving a positive outcome). An early study in remitted AN reported a decrease in ability to learn implicit categories following a rule change [50]; the researchers conducted simulations and mathematical modeling of cognitive processes to identify what may give rise to enhanced initial learning and difficulties adapting after a rule change. Results from these models suggested that one explanation for the AN groups’ behavior was that an increased sensitivity to punishment facilitated adaptive learning of initial contingencies, but that difficulties with flexibility and rule selection accounted for deficits once the rules of the task changed. Enhanced learning from punishment and increased likelihood of shifting behavior after losing (i.e., lose-shift) has also been observed in acutely ill adolescents [49] and remitted adults [28, 53]. However, results regarding performance on reversal learning tasks have remained conflicting, with some studies indicating that individuals remitted from AN have enhanced ability to adapt learning rates to volatile, or changing, environments [54], and other studies finding that those with AN have no differences in performance on reversal learning tasks compared to control subjects [55, 56].

### Fear and Avoidance Learning in AN

Early conceptualizations of AN emphasized fear-related symptoms hypothesized to result from pathological fear conditioning [57]. However, only a few studies have assessed fear learning in AN, and most assessed conditioning to an aversive human scream. These studies suggest no abnormalities in fear conditioning among adolescents [58] or adults [59] with AN, though adults reported greater threat expectancies during extinction and renewal using a Likert scale. Modern theories have shifted away from fear conditioning as a mechanism of AN towards avoidance models that propose eating disorder behaviors function as a means of avoiding feared aversive outcomes such as weight gain, anxiety associated with eating or gastric distress [60]. Notably, while the ability to learn how to avoid harm is critical for health and survival, excessive avoidance learning can lead to chronic maladaptive avoidance or compulsive behaviors. Harm avoidance learning models tend to focus on aversive physical or interoceptive (e.g., body-state) experiences, which have relevance for eating disorders [61–63]; thus, we and others are beginning to examine reinforcement learning performance in the context of aversive interoceptive outcomes.

### Habit and Goal-directed Learning in AN

The capacity to arbitrate between goal-directed and habit learning systems is necessary for flexible behavior; AN has been theorized to reflect extreme goal pursuit (e.g., weight loss) with dietary restriction reflecting inflexible, repetitive, or compulsive behavior that is initially maintained by instrumental learning (e.g., reinforced by reward of weight loss and praise) that becomes a classically conditioned habit over time [26, 64, 65]. However, while there is emerging evidence to suggest food choice, or the tendency to choose low-calorie over high-calorie foods, is habit-driven [29, 66], few studies have directly examined habit and/or goal-directed reinforcement learning in AN, and existing findings are mixed. Studies using tasks evaluating the persistence of devalued actions (slip-of-action task [67]) report intact goal-directed learning but no differences in habitual responding between AN, remitted AN, or healthy controls [65, 68]. Alternatively, a study that used a two-step sequential decision-making task that can be computationally modeled to dissociate model-based (goal-directed) and model-free (habit) reinforcement learning [23, 69–71] demonstrated attenuation in model-based reward learning across food- and non-food related learning in individuals with full-threshold AN [25], consistent with the proposal that enhanced reward-based, habit-learning may support the maintenance of AN [72].

In summary, there is growing evidence supporting altered reinforcement learning in AN. Specifically, poorer reward- and punishment-based feedback learning appear to be associated with acute illness state and are not consistently observed in remitted samples. Indeed, some evidence suggests faster punishment learning in remitted samples compared to controls [46] and acutely ill individuals (unpublished data), raising the question of whether reward and punishment learning are biomarkers of illness persistence or are modifiable factors to promote recovery. Moreover, initial studies support the role of decreased goal-directed learning in AN, although these findings need to be replicated. Similarly, additional research must resolve mixed findings related to learning under changing rules. Lastly, while there is limited evidence of altered fear conditioning (though subjective threat expectation may be heightened; [59, 64]), studies of avoidance learning may help to distinguish between loss-related learning (e.g., learning to change a behavior by experiencing a negative consequence) and learning to avoid aversive negative consequences in AN (e.g., learned actions that prevent a negative outcome from occurring). Recent methodological advances may improve understanding of reinforcement learning and decision-making in AN.

## Computational Modeling Can Provide a More Nuanced Understanding of Mechanisms Driving Behavior to Better Characterize Reinforcement Learning and Decision-making in AN

Traditional experimental approaches have operationalized “learning” using observed behavioral choices, such as the percentage of accurate choices made or the number of times an individual opted to stick with the same choice following positive feedback (win-stay behavior) or change choices following negative feedback (lose-shift behavior), which have suggested altered performance in AN. Computational modeling approaches utilize mathematical equations to quantify hypothetical latent cognitive and/or neural processes contributing to observed choice behavior and can provide a richer mechanistic understanding of learning and decision-making [73, 74]. More specifically, researchers compare the simulated performance on a given task using a hypothesized model of interacting cognitive processes to the performance of human participants. Within a given model, researchers can estimate the values of different paramters—or hypothesized cognitive or neural contributors to performance—for a given individual that best account for their perforamnce within a given model framework, providing an account of individual differences in subcomponents of the learning process. In sum, researchers can use the relative fits of competing computational models to deduce potential latent processes that are contributing to observed behavior; they can also use estimated parameter values for participants and groups within a well-fitting model to draw tentative conclusions about individual or group differences in how a specific latent process may vary or relate to symptoms.

Temporal difference (TD) models are the most commonly-used equations to estimate instrumental reinforcement learning and reflect the core idea that learning occurs through updating the estimated value of the current “state” (environment or cues that a person is exposed to) or potential actions based on prediction errors (i.e., the difference between the predicted value of the current state or action and the actual value) on a trial-by-trial basis. There are different computational methods for updating the value of the current state-action pair; the SARSA (state-action-reward-state-action) method bases this on the action actually taken, whereas the Q-learning method bases this on the best possible action in the next state [17]. TD models only incorporate choice behavior (e.g., which action was selected) in their algorithms, but do not explain how decisions are made over time. Drift-diffusion model algorithms represent another commonly-used decision-making model that hypothesizes several processes that contribute to reaction times across each trial. This sequential sampling approach explains how decisions are made as a result of reaching a threshold of accumulating evidence over time [17]. For example, we recently fit a hybrid Q-learning TD and drift-diffusion model of associative learning to examine both learning and decision-making processes in adults remitted from AN [46]. We found that individuals remitted from AN showed better learning from negative PE than controls, suggesting an enhanced ability to learn from outcomes that were worse than expected. Moreover, parameters derived from the drift-diffusion model indicated that the remitted AN group demonstrated a reduction in accuracy of optimal choices and rate of information extraction on reward trials as the task progressed, suggesting that their efficiency in making reward-related decisions decreased over the task, perhaps secondary to increased attention toward negative outcomes. This raises the hypothesis that better learning from negative, rather than positive, feedback in remitted AN may relate to recovery. However, longitudinal studies are needed to determine how changes in reward and punishment learning relate to symptom persistence or remission.

### Individual Model Parameter Estimates Could Reveal Underlying Processes Contributing To Overall Performance

While computational models of cognition rely on assumptions about overall cognitive performance, to understand actual human behavior it is necessary to identify a set of individual parameters that can predict empirical observations, a process known as model-fitting. As noted above, individual model parameters, which can be varied to fine-tune the model’s functioning, determine how observed data are related to latent cognitive processes, and can provide nuanced information about individual or group differences in processes contributing to performance [75]. Learning rates are the most frequently reported model parameters in learning studies; however, other model parameters may also have relevance to AN. For instance, the explore-exploit tradeoff, reflected by the inverse temperature parameter (β) within temporal difference learning algorithms, has been proposed to explain maladaptive decision-making in eating disorders [76]. In the context of value-based decision-making and reinforcement learning, exploration involves sampling a wide range of options, including those with lower likelihood of reward, to discover more valuable options, whereas exploitation involves repeatedly choosing a known or previously rewarded option, which requires less cognitive resources but may result in prematurely settling on a suboptimal solution before gathering sufficient information [17]. Studies of foraging behavior, which assess goal pursuit by evaluating decisions to accept a current reward or not without knowing the other rewards available [77], have implicated the explore/exploit tradeoff in psychopathology. For instance, attention-deficit/hyperactivity disorder symptoms and obsessive-compulsive symptoms have been associated with random exploration [78], and substance use disorders are associed with excessive exploitation [79, 80]. Notably, in eating disorders, preliminary evidence suggests adults with bulimia nervosa were more exploratory than healthy controls on a patch-foraging task [76], which was interpreted to suggest that excessive exploration could promote impulsive behaviors characteristic of bulimia nervosa. In contrast, we observed greater exploration (smaller β values) in adults with AN compared to healthy controls during a probabilistic associative learning task for reward and punishment [27]. We interpreted this strategy of exploring more than exploiting stimulus-response-outcome hypotheses to indicate that when ill, adults with AN may less decisively make choices and continue to explore stimulus-response outcomes rather than employing the same strategy across all trials. Diminished certainty in exploiting what they learned may reflect their uncertainty in the task contingencies given that AN is often characterized by increased sensitivity to uncertainty [81], although this was not directly tested. Together, these findings highlight the potential value in examining individual model parameters. However, computational findings to date have rarely been linked with illness symptoms, and current investigations remain limited in scope given the large number of parameters that can be investigated, both across groups and in association with symptoms to provide insight into individual differences.

### Integration of Model Parameter Estimates may Support Novel Approaches to Grouping Individuals to Inform Precision Medicine

The sum may be greater than the parts; in addition to evaluating model parameter estimates individually, examining parameters in combination may reveal phenotypic patterns of performance that can inform precision medicine. Precision medicine aims to personalize treatment based on individual characteristics thought to underlie disease, with the goal of achieving better outcome than traditional one-size-fits-all treatment approaches. By focusing on individual variability in cognition, neural function and symptom expression, computational modeling of cognition may guide precision medicine by providing a framework to identify subgroups who share biological mechanisms of psychiatric symptoms [82]. Recent work has demonstrated innovative ways of classifying individuals on the basis of reinforcement learning performance or comparing reinforcement learning across data-determined groups. For example, in a transdiagnostic sample of individuals with obsessive compulsive disorder, social anxiety disorder, and AN, a latent class analysis identified four groups based on unique psychophysiological patterns of fear acquisition and extinction learning. Notably, these data-driven groups did not align with traditional DSM-5 diagnostic classification, but the groups were differentially associated with dimensional measures of anxiety [83]. Another study identified two subgroups in AN characterized by goal-directed and habit-driven behavior based on responses to a self-report measure; differences in neural activation during a separate reward anticipation task were only observed when these groups were examined separately, with lower medial orbitofrontal cortex (OFC) activity in the habit-driven group consistent with a rigid behavioral approach [84]. A third study in adults with AN and bulimia nervosa that classified groups based on personality, with an “undercontrolled” group characterized by high levels of impulsivity and dysregulation, an “overcontrolled” group characterized by high levels of rigidity and avoidance, and a “low psychopathology” group characterized by relatively normal levels of personality functioning, reported elevated reward responsiveness and poorer reversal learning performance in the “low psychopathology” group, suggesting that elevated drive for rewards and difficulty adjusting to changing reward contingencies may contribute to persistence of illness for this particular class [85]. Collectively, these studies support novel approaches that may improve precision in differentiating groups based on individual characteristics, such as reinforcement learning, that may relate more specifically to symptom expression and could inform personalized treatment.

## Future Directions

Despite recent advances in characterizing reinforcement learning in AN, there remain several areas in need of further study, including extending paradigms to examine other contextual factors, evaluating longitudinal changes in these processes, and elucidating the neural substrates of reinforcement learning to inform biological models. For instance, reinforcement learning tasks typically use symbolic feedback and secondary reinforcers such as monetary gains and losses as outcomes; however, the salience or generalizability of these outcomes has been questioned in light of evidence suggesting there may be differences in processing and learning from disorder-specific stimuli (e.g., food pictures, thin models) vs. general stimuli (e.g., money) [38, 86]. Notably, while one study that assessed both monetary and “snack point” outcomes reported that individuals with AN showed worse model-based, but not model-free, learning in both conditions, suggesting a general imbalance in habitual vs. goal-directed control across domains [25], another study found that reversal learning deficits only emerged in AN in food-related, and not neutral, contexts [87]. Thus, questions remain regarding the specificity of reinforcement learning alterations in AN and possible impacts of other disorder-relevant cues and outcomes (e.g., social outcomes, interoceptive sensations). Moreover, while theoretical models posit that altered reinforcement learning contributes to the onset (e.g., symptoms emerge as the result of maladaptive learning) and maintenance (e.g., symptoms persist because maladaptive learning interferes with an individuals’ ability to change) of psychiatric disorders [10, 88–90], longitudinal studies are needed to characterize the role of reinforcement learning in illness trajectory, especially given that rapid changes in reinforcement learning in adolescence coincide with the developmental period of AN onset [91]. Notably, emerging evidence in other areas of psychiatry implicates longitudinal change in reinforcement learning with treatment outcome; improvement in reinforcement learning performance has been associated with continued abstinence following tobacco cessation [92] and with maintenance of remission in bipolar disorder [93]. There have been few longitudinal reinforcement learning studies in AN. One study reported no changes in model-free or habit-based learning following weight restoration [25]. Another study reported that poorer reversal learning predicted an increase in purging over 6 months in a combined sample of individuals with AN binge-eating/purging subtype and bulimia nervosa, but longitudinal associations with eating disorder symptoms in AN restricting type were not detected [55].

Lastly, while the neural circuits underlying reinforcement learning and decision-making are well delineated in healthy controls, they remain poorly understood in AN. Human fMRI studies support the striatum as the key brain area for encoding PEs during reinforcement learning [94], with dorsal functional divisions supporting feedback learning and ventral regions encoding the valence of events (reward/punishment learning) [95]. Learning also relies on cortico-striatal connectivity, including with the medial orbitofrontal cortex (mOFC; involved in integrating inferred outcomes with task structure) [96] and the prefrontal cortex (PFC), including the ventromedial PFC (vmPFC; involved in outcome value representation) [23] and dorsolateral PFC (dlPFC; involved in sequence action planning and associative learning). Neuroimaging studies of reversal learning in AN have reported increased activity in the medial PFC during acquisition of a food-cue association and its reversal [97], and during negative feedback trials [28, 49, 98], suggesting greater recruitment of cognitive control neurocircuitry in AN. Systematic characterization of neural circuit disruptions corresponding to behavioral learning differences in AN may inform treatment targets for biologically-based treatments, including pharmacological or neuromodulatory approaches.

## Clinical Implications

Alterations in reinforcement learning in AN may provide an account of symptom persistence, providing insights that can inform adapted, novel interventions. Many of the existing first-line psychological therapies are based on cognitive-behavioral frameworks and principles of learning theory [99]. Prevailing cognitive-behavioral models conceptualize thoughts, behaviors, and emotions as learned responses that can be altered through techniques that promote new or corrective associative learning by providing experiences that facilitate updating of prior associations with food and weight-related cues (i.e., extinction learning) or forming new adaptive competing associations (i.e., inhibitory learning) [100, 101]. For example, through approaching previously avoided foods, individuals can learn through experience that food consumption does not necessarily result in the feared outcome, or that feared outcomes are tolerable and/or transient, countering learned food avoidance. However, because these treatments presuppose that individuals can update old learning and engage in new learning, individual differences in learning (e.g., difficulty updating prior learning, extreme bias towards reward or punishment learning) may interfere with therapeutic change [102]. Research in other fields has suggested that behavioral interventions can be effectively personalized to maximize learning among those with learning differences; therefore, understanding reinforcement learning in AN could inform the development of personalized behavioral interventions.

## Conclusions

Emerging evidence supports the role of altered reinforcement learning in AN. Currently, most studies have examined the influence of outcome valence, changing rules and uncertainty, and habitual vs. goal-directed control on instrumental learning in samples of adolescents and adults either ill or remitted from AN. In addition to observed performance, computational modeling of latent cognitive processes underlying reinforcement learning shows promise in detecting nuanced behavioral differences that may relate more precisely to AN symptomatology and inform personalized treatments. Future research is needed to identify which aspects of reinforcement learning are altered in an individual, and how these individual differences in associative learning at baseline, or over time, may predict outcomes (see [103]).

## Key References

- Brown CS, Nunez A, Wierenga CE. Altered value-based decision-making in anorexia nervosa: A systematic review. Neurosci Biobehav Rev. 2024;167:105944.
  - (This systematic review provides a comprehensive overview of the value-based decision-making literature in AN, and summarizes studies according to illness state, developmental stage, and AN subtype.)

- Foerde K, et al. Deficient goal-directed control in a population characterized by extreme goal pursuit. J Cogn Neurosci. 2021;33(3):463–81.
  - (This is the first study to examine model-free and model-based learning in AN, and demonstrates attenuated model-based reward learning for money and snack points in adults with AN, suggesting increased habitual control.)

- Garcia-Burgos D, et al. Food restriction in anorexia nervosa in the light of modern learning theory: A narrative review. Behav Sci (Basel). 2023;13(2).
  - (This narrative review provides a comprehensive overview of modern learning theory applied to AN and a review of relevant research studies.)

- Melles H, Spix M, Jansen A. Avoidance in anorexia nervosa: Towards a research agenda. Physiol Behav. 2021;238:113478.
  - (This theoretical paper proposes a framework supporting the role of avoidance learning in AN and puts forth a research agenda including basic and clinical experimental research to elucidate avoidance mechanisms thought to maintain illness.)

- Murray SB, et al. A multi-modal assessment of fear conditioning in adolescent anorexia nervosa. Int J Eat Disord. 2024;57(7):1499–1509.
  - (This study shows that fear acquisition and extinction do not differ in adolescent girls with AN and healthy peers, suggesting no abnormalities in fear learning in adolescents with AN).

- Pearce AL, Fuchs BA, Keller KL. The role of reinforcement learning and value-based decision-making frameworks in understanding food choice and eating behaviors. Front Nutr. 2022;9:1021868.
  - (This theoretical paper discusses the role of reinforcement learning in eating behaviors from different perspectives including sign-versus-goal-tracking phenotypes, a model-free versus model-based framework, and the utility or value-based model.)

- Pike AC, et al. Adaptive learning from outcome contingencies in eating-disorder risk groups. Translational Psychiatry. 2023;13(1):340.
  - (This study shows that individuals remitted from AN are better able to adjust learning when outcome contingencies change compared to healthy peers.)

- Radzikowska M, Pike AC, Hall-McMaster S. Computational perspectives on cognition in anorexia nervosa: A systematic review. Comput Psychiatr. 2025;9(1):100–21.
  - (This systematic review examines the computational literature in AN, highlighting studies related to reinforcement learning, value-based decision-making, goal-directed and habitual control over behavior, cognitive flexibility, and theory-based accounts.)

- Reilly EE, Wierenga CE, Le Grange D. Testing the role of associative learning in evidence-based treatments for anorexia nervosa. Int J Eat Disord. 2024;57(5):1088–95.
  - (This manuscript proposes a conceptual framework for evaluating the contribution of associative learning to existing psychological treatments for AN.)

- Schaefer L, Steinglass J. Reward learning through the lens of RDoC: A review of theory, assessment, and empirical findings in the eating disorders. Curr Psychiatry Rep. 2021;23(1):2.
  - (This manuscript reviews theories, behavioral and self-report assessments, and empirical findings related to reward learning in eating disorders using the NIMH Research Domain Criteria as a guide).

- Uniacke B, et al. Altered learning from positive feedback in adolescents with anorexia nervosa. J Int Neuropsychol Soc. 2024;30(7):651–59.
  - (This study demonstrates that adolescents with AN have a significantly lower rate of learning from positive feedback relative to healthy peers.)

- Wierenga CE, et al. Altered reinforcement learning from reward and punishment in anorexia nervosa: Evidence from computational modeling. J Int Neuropsychol Soc. 2022;28(10):1003–15.
  - (This study demonstrates lower rates of learning following both positive and negative outcomes in adults with AN and shows that lower punishment learning is associated with less weight gain during treatment, implicating reinforcement learning in treatment response.)

- Wierenga CE, et al. Reinforcement learning in women remitted from anorexia nervosa: Preliminary examination with a hybrid reinforcement learning/drift diffusion model. J Int Neuropsychol Soc. 2025;1–11.
  - (This study demonstrates higher rates of learning following negative outcomes in adults remitted from AN, implicating state and trait effects on reinforcement learning.)

## Data Availability

No datasets were generated or analysed during the current study.
